# Automatic Segmentation and Measurement on Knee Computerized Tomography Images for Patellar Dislocation Diagnosis

**DOI:** 10.1155/2020/1782531

**Published:** 2020-01-28

**Authors:** Limin Sun, Qi Kong, Yan Huang, Jiushan Yang, Shaoshan Wang, Ruiqi Zou, Yilong Yin, Jingliang Peng

**Affiliations:** ^1^School of Software, Shandong University, Jinan, Shandong 250101, China; ^2^State Grid Anhui Electric Power Company, Hefei, Anhui 230061, China; ^3^First Affiliated Hospital of Shandong University of Traditional Chinese Medicine, Jinan, Shandong 250014, China

## Abstract

Traditionally, for diagnosing patellar dislocation, clinicians make manual geometric measurements on computerized tomography (CT) images taken in the knee area, which is often complex and error-prone. Therefore, we develop a prototype CAD system for automatic measurement and diagnosis. We firstly segment the patella and the femur regions on the CT images and then measure two geometric quantities, patellar tilt angle (PTA), and patellar lateral shift (PLS) automatically on the segmentation results, which are finally used to assist in diagnoses. The proposed quantities are proved valid and the proposed algorithms are proved effective by experiments.

## 1. Introduction

Patellar dislocation occurs when the patella slips out from the patellar surface of the femur. It is a common knee injury that may happen when people, especially teenagers and athletes, do vigorous physical exercises, *e.g.*, playing basketball and football. To help the diagnosis, computerized tomography (CT) images are often taken at the knee area. On the knee CT images, clinicians usually make manual measurements and make diagnosis according to the measured results. The manual measurement is often complex, tedious, and error-prone. Therefore, a fully automatic approach by computers is highly wanted.

Computed tomography has been widely used to diagnose knee joint pathologies. Correspondingly, knee CT images have been automatically or semiautomatically processed and analyzed (*e.g.*, [1–5]) for computer-aided diagnosis. Subburaj et al. [1] proposed a computer graphics-based method to automatically localize and label anatomical landmarks on the 3D bone model reconstructed from knee CT images of a patient. Krcah et al. [2] proposed to segment the femur in 3D CT volumes based on graph cuts and a bone boundary enhancement filter. Jang et al. [3] compared and validated various segmentation algorithms to segment the knee CT images and construct a corresponding 3D model. Wu et al. [4] proposed to segment multiple bones around the knee joint with severe pathologies to help patient-specific orthopedic knee surgery planning. Mezlini et al. [5] proposed to measure the knee joint space based on semiautomatic CT image segmentation for the monitoring of osteoarthritis progression. However, to the best of our knowledge, no efforts have been published specifically for automatic measurement on knee CT images for the purpose of patellar dislocation diagnosis.

The major contributions of our work reside in the following aspects. Firstly, we propose two quantities, patellar tilt angle (PTA) and patellar lateral shift (PLS), to measure on the knee CT images. Secondly, in order to make the automatic measurement, we propose computing algorithms to segment the patella and femur regions in the CT images and measure the proposed quantities on the segmented regions. Finally, we make experiments to verify the validity and effectiveness of the measured results for the computer-aided diagnosis (CAD). Note that a preliminary version of our work has been published in reference [6]. Extending the preliminary work [6], we utilize the correlation between adjacent CT images by bone region prediction for bone region segmentation and make more complete experimental validation in terms of accuracy of measurement and applicability for CAD in this work.

## 2. Scheme Overview

The proposed scheme takes a specific portion of knee CT images as input and conducts a complete and automatic process of bone regions segmentation and geometric measurement.

### 2.1. Input Images

**Figure 1:**
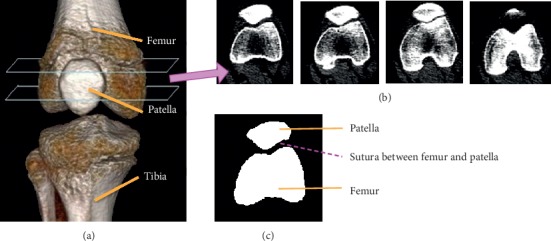
Examples of input CT images and segmentation result. We use as input the CT images of the cross sections cutting through the femur and the patella, as delimited by two blue parallelograms in (a). Examples of the input CT images are shown in (b), and an ideal segmentation result on a CT image is shown in (c).

The source CT images for a patient are acquired by scanning the middle part of his or her leg. While being scanned, the patient may move his or her leg naturally through a range of knee angles, resulting in multiple sequences of CT images sampled at a preset temporal frequency. For each image sequence acquired at a time instance, we only use a portion that corresponds to cross sections through the femur and the patella. As an example, the anatomical structure of the middle part of a leg is shown in Figure 1(a) with the femur, patella, and tibia labelled. As shown in Figure 1(a), the portion of CT images that we use corresponds to the cross sections between the two planes as marked with blue parallelograms. Examples of the CT images in this portion are shown in Figure 1(b), and the ideal segmentation result for a CT image is shown in Figure 1(c) where the femur and the patella regions are neatly segmented and the narrow gap between them corresponds to the sutura.

We presume that the input CT images are ordered such that images of higher scanning positions on the leg go earlier in the sequence.

### 2.2. Working Process

**Figure 2:**
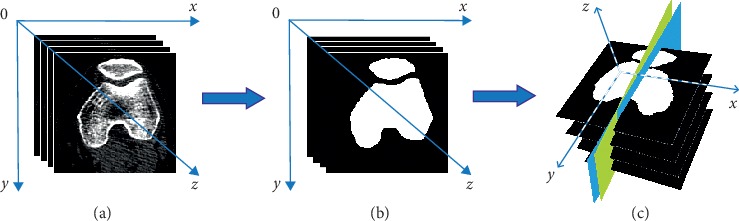
Illustration of the working process: (a) input CT images, (b) patella and femur region segmentation, and (c) central plane fitting and geometric measurement.

We use Figure 2 to illustrate the automatic segmentation and measurement process. For an input CT image sequence as shown in Figure 2(a), we first segment the femur and the patella regions on each image to get the result as shown in Figure 2(b). Based on the profiles of the segmented regions, we use least squares fitting to find the central planes of the femur and the patella, respectively, as shown in Figure 2(c). Finally, we quantify the geometric relationship between the two central planes by PTA and PLS, which provide the basis for patellar dislocation diagnosis.

## 3. Segmentation of Femur and Patella Regions

**Figure 3:**
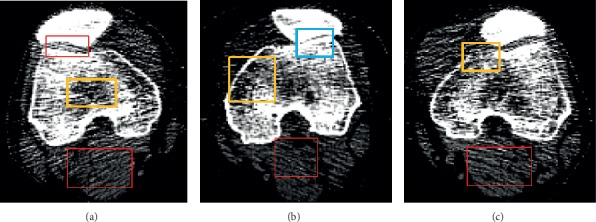
Challenging characteristics of knee CT images, as illustrated by the examples in (a), (b), and (c). The red rectangles mark the responses of soft tissues and noises and the yellow rectangles mark intensity inhomogeneity while the blue rectangle marks the narrow sutura region between the femur and the patella.

Segmenting two solid bone regions corresponding to the femur and the patella, respectively, in each knee CT image is a key step in our scheme. It is a challenging task due to the following characteristics of knee CT images (as illustrated by Figure 3): (1) a single CT image usually contains responses of bones and other tissues (*e.g.*, soft tissues) simultaneously and is contaminated with noises; (2) the patella and the femur regions may be very close to each another (*e.g.*, only a couple of pixels apart or even locally fused) in many CT images; and (3) different parts (*e.g.*, cortical bone tissue, spongy bone tissue, bone marrow, and bone cavity) inside a bone usually have different radiological densities, leading to highly variant gray levels of pixels in one bone region.

The generic problem of image segmentation has been researched for decades. For a survey of the early-days algorithms, we refer to references [7, 8]. Later on, with the development of medical imaging technology, intensive and specific efforts have been made to segment various types of medical images. The existent medical image segmentation algorithms can be classified as threshold-based methods [2], region-based methods [9–11], edge-based methods [12], active-contour-model-based methods [13–22], hybrid methods [23–26], and others [27–32].

Among the various methods, the active-contour-model-based ones appear more advantageous to us. Relatively speaking, they handle structures with high topological complexity well and achieve subpixel accuracy and robustness against noise. In addition, they incorporate easily with other segmentation techniques and facilitate intuitive interaction [33, 34]. In particular, we choose the Chan–Vese (C-V) region-based active contour models [17] for our knee CT image segmentation, as it is in general less sensitive to initialization and noise than many other methods [13–19] of its category. Further, according to our experiments (see Section 5), it yields better segmentation results than the other selected active-contour-model-based methods [18, 21, 22], when used with our proposed framework.

The existent image or medical image segmentation algorithms usually assume that the pixels inside a meaningful region have highly uniform levels of intensity. In addition, the contrast between meaningful and nonmeaningful regions and the noise level in an image also influence the segmentation results. These algorithms cannot be directly applied for our purpose due to the highly challenging characteristics of the knee CT images, as described earlier in the text. Therefore, we propose to improve the quality of the knee CT images first, by increasing the uniformity of pixel intensities, enhancing the contrast, and suppressing the noises, before making the final segmentation. Specifically, we process the CT images in an input sequence one by one in spatial order. For each CT image, we enhance its contrast to increase (decrease) the gray levels of the bone tissue (soft tissue and noise) pixels, predict the bone and sutura regions in it and modify its pixel values accordingly, utilizing the segmentation result of the previously processed CT image, if any, and employ the C-V region-based active contour method to make the final segmentation on the modified image. Details of these steps are given in the following sections.

### 3.1. Contrast Enhancement

**Figure 4:**
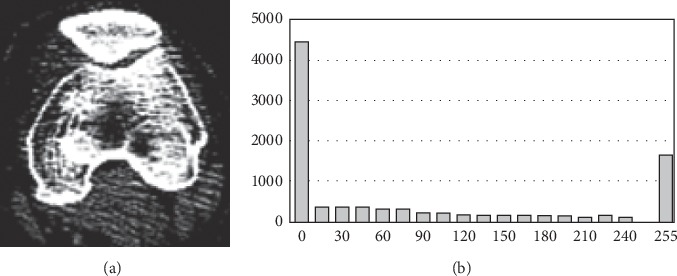
(a) A knee CT image and (b) the histogram of (a). The histogram shows that the pixels' gray levels concentrate around very low and very high values.

The common global contrast enhancement method based on histogram manipulation does not work well for our case. The reason is that the pixels' gray levels concentrate around very low and very high values (see Figure 4), leaving little room for contrast enhancement. Instead, we propose a contrast enhancement method based on local characteristics around each pixel. Observing that higher (lower) gray levels correspond to bone tissues (soft tissues and probably noise), we increase (decrease) the gray level of a pixel with brighter (darker) neighborhood.

Specifically, we perform a nonlinear scaling of each pixel's gray level according to its neighboring pixels' gray levels [6]. For a pixel, *p*_0_, we denote its gray level as *g*_0_ and the gray levels of all the other pixels in *p*_0_'s 3 × 3 neighborhood as *g*_*i*_ (*i* ∈ 1,2,…, 8). Assuming that the maximum gray level is 255, we update *g*_0_ to *g*_0_′ according to

(1) $$\begin{array}{c} g_{0}^{\prime }=g_{0}\times e^{\alpha -0.45},\\ \alpha =\frac{{\sum_{i=0}^{8}g_{i}}^{}}{255\times 9}. \end{array}$$

We find by experiments that the above process, when iterated for two or three passes, yields good results.

**Figure 5:**
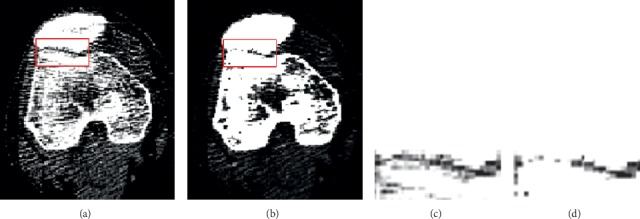
Effect of the contrast enhancement. (a) Original CT image, (b) contrast-enhanced image, and (c, d) the enlarged view of the corresponding sutura areas in (a) and (b), respectively.

We show the effect of contrast enhancement on an example CT image in Figure 5, where the original image and the contrast-enhanced image are shown in Figures 5(a) and 5(b), respectively, and the enlarged views of the corresponding sutura areas are shown in Figures 5(c) and 5(d), respectively. Comparing Figures 5(a) and 5(b), we see that the bone pixels are emphasized while the soft tissue and noise pixels are suppressed in general. Comparing Figures 5(c) and 5(d), however, we find that the intensity of some pixels in the sutura region is unwantedly increased at the same time, reducing the gap between two bone regions and adding to the difficulty of bone regions segmentation. This issue is addressed by the proposed bone regions prediction technique, as described in the following section.

### 3.2. Prediction of Bone Regions

Narrow and vague gap between bone regions and inhomogeneous pixel intensity within bone regions are limiting factors for accurate bone region segmentation. In order to address these issues, we propose a bone region prediction process that further improves the CT image quality to facilitate accurate segmentation, as detailed below.

On any input CT image sequence used in our experiments, we observe two facts: firstly, the femur and the patella regions are relatively small and wide apart and contain highly homogeneous pixel intensity in the initial CT images of the sequence; secondly, the shape and the position of a bone's profile vary only slightly between two adjacent CT images in the sequence. The former implies that we may apply a prevalent image segmentation algorithm on the first CT image (after contrast enhancement) in the sequence to obtain a good result, while the latter implies that a good segmentation result on a CT image may be utilized to predict the bone and sutura regions in the next image to be segmented.

Assume that we are currently processing the (*n*+1)-th (*n* ≥ 0) original CT image, *I*_ori_^*n*+1^, in the sequence. After the contrast enhancement, we obtain *I*_enh_^*n*+1^. If *n*=0, we simply use *I*_enh_^*n*+1^ as the modified image, *I*_mod_^*n*+1^, which is to be segmented. Otherwise, we already have the segmentation result for the *n*-th image, which is a binary image, *I*_seg_^*n*^, with “255”-pixels for the bone regions and “0”-pixels for the background. Using *I*_seg_^*n*^, we improve the quality of *I*_ori_^*n*+1^ by a proposed process of bone regions prediction to obtain *I*_mod_^*n*+1^, as detailed below.

**Figure 6:**
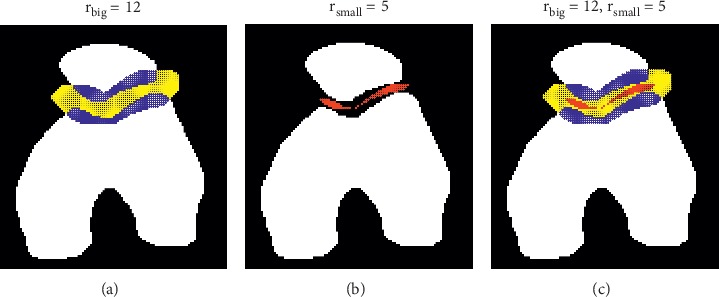
Predictions of various local regions around the sutura using a sample segmentation image. As shown in (a), the predicted sutura region (*Q*_1_) and local bone region around (*Q*_2_) are colored in yellow and blue, respectively. As shown in (b), the predicted thin pixel layer (*B*) between the bone regions is colored in red. An overlaid visualization of all these predicted local regions is given in (c). We use *r*_big_=12 and *r*_small_=5 when constructing these local regions.

Firstly, based on *I*_seg_^*n*^, we predict in *I*_ori_^*n*+1^ the sutura region, *Q*_1_, and the local bone region, *Q*_2_, around the sutura, enabling us to treat these local regions with special care in the following steps. Specifically, in *I*_seg_^*n*^, we morphologically dilate the femur region, *F*^*n*^, and the patella region, *P*^*n*^, to *F*_*d*_^*n*^ and *P*_*d*_^*n*^, respectively, by a disk with a radius of *r*_big_ pixels. Empirically, we take *r*_big_ ∈ [8,12]. Locations in *Q*=*F*_*d*_^*n*^∩*P*_*d*_^*n*^ which correspond to nonbone pixels (“0”-pixels) in *I*_seg_^*n*^ form the predicted sutura region, *Q*_1_, and *Q*_2_=*Q* − *Q*_1_ gives the predicted local bone region around the sutura in *I*_ori_^*n*+1^. An example of the local regions prediction is shown in Figure 6(a) with *Q*_1_ and *Q*_2_ colored in yellow and blue, respectively.

Secondly, we selectively revert pixels in *I*_enh_^*n*+1^ which fall inside *Q* by *I*_enh_^*n*+1^(*x*, *y*)=*I*_ori_^*n*+1^(*x*, *y*), (*x*, *y*) ∈ *Q*. This is based on the observation that the contrast enhancement tends to narrow the gap between the two bone regions (see Figures 5(c) and 5(d)), adding to the difficulty of bone regions segmentation.

Thirdly, in order to increase the bone regions' density homogeneity, we combine *I*_enh_^*n*+1^ with *I*_seg_^*n*^ to obtain *I*_mod_^*n*+1^ according to

(2) $$\begin{array}{c} I_{\text{mod}}^{n+1}\left(x,y\right)=\left\{\begin{array}{cc} \alpha I_{\text{seg}}^{n}\left(x,y\right)+\beta I_{\text{enh}}^{n+1}\left(x,y\right), & I_{\text{enh}}^{n+1}\left(x,y\right)<{\text{th}}_{a},\\ I_{\text{enh}}^{n+1}\left(x,y\right), & \text{otherwise,} \end{array}\right. \end{array}$$

where *α*, *β*, and th_*a*_ are parameters to control the degree of fusion. We empirically use *α*=0.4, *β*=0.6, and th_*a*_=0.5 × 255. In extreme cases, if th_*a*_=0, *I*_mod_^*n*+1^=*I*_enh_^*n*+1^ and if th_*a*_=256, *I*_mod_^*n*+1^=*αI*_seg_^*n*^+*βI*_enh_^*n*+1^.

Fourthly, we reduce the intensities of the predicted local region, *Q*, around the sutura in *I*_mod_^*n*+1^, making a clearer separation of the two bone regions. This is achieved by selectively updating pixels in *I*_mod_^*n*+1^ according to

(3) $$\begin{array}{c} I_{\text{mod}}^{n+1}\left(x,y\right)=\left\{\begin{array}{cc} \mu_{1}I_{\text{mod}}^{n+1}\left(x,y\right), & \left(x,y\right)\in Q_{1}\wedge I_{\text{ori}}^{n+1}\left(x,y\right)<{\text{th}}_{b},\\ \mu_{2}I_{\text{mod}}^{n+1}\left(x,y\right), & \left(x,y\right)\in Q_{2}\wedge I_{\text{ori}}^{n+1}\left(x,y\right)<{\text{th}}_{b}, \end{array}\right. \end{array}$$

where th_*b*_ is a threshold set to the mean gray level of all our test CT images, and we empirically use *μ*_1_=0 and *μ*_2_=0.5. By equation (3), we weaken pixels in *Q* whose original intensities are below a threshold. If a pixel's original intensity is above the threshold, however, it is probably a bone pixel and we leave it untouched. Note that we weaken pixels in two subregions, *Q*_1_ and *Q*_2_, since the predicted sutura region, *Q*_1_, is usually not completely precise and we choose to weaken selected pixels in a wider local area, *i.e.*, *Q*_1_+*Q*_2_.

Lastly, based on *I*_seg_^*n*^, we predict in *I*_mod_^*n*+1^ a thin layer, *B*, of pixels between the two bone regions when they get close to each other and set these pixels to “0” for further separation of the bone regions. Specifically, in *I*_seg_^*n*^, we morphologically dilate *F*^*n*^ and *P*^*n*^ by a disk with a radius of *r*_small_ pixels to *F*_*d*_^′*n*^ and *P*_*d*_^′*n*^, respectively, and obtain *B*=*F*_*d*_^′*n*^∩*P*_*d*_^′*n*^. Empirically, we take *r*_small_ ∈ [4,6]. An example is shown in Figure 6(b) with *B* colored in red. Depending on the shapes of and the distance between the two bones regions, there may be none, one, or multiple connected components in *B*.

### 3.3. C-V Region-Based Active Contour Segmentation

After *I*_ori_^*n*+1^ (*n* ≥ 0) is modified to *I*_mod_^*n*+1^, we employ the C-V models to segment *I*_mod_^*n*+1^. The C-V model was originally proposed by Chan and Vese [17] and is based on the following energy model:

(4) $$\begin{array}{c} F^{CV}\left(c_{1},c_{2},C\right)=\lambda_{1}\int_{C_{\text{in}}}{\left|I\left(x\right)-c_{1}\right|}^{2}\text{d}x+\lambda_{2}\int_{C_{\text{out}}}{\left|I\left(x\right)-c_{2}\right|}^{2}\text{d}x+\mu \cdot \text{Length}\left(C\right)+\nu \cdot \text{Area}\left(\text{inside}\left(C\right)\right), \end{array}$$

where *λ*_1_, *λ*_2_, *µ*, and *ν* are constants, *C*_in_ and *C*_out_ represent the regions inside and outside contour *C*, respectively, and *c*_1_ and *c*_2_ correspond to the average pixel intensity in *C*_in_ and *C*_out_, respectively. The solution of optimal contour *C* is reached by minimizing the energy function *F*^*CV*^(*c*_1_, *c*_2_, *C*), resulting in an optimal segmentation of the image *I*.

**Figure 7:**
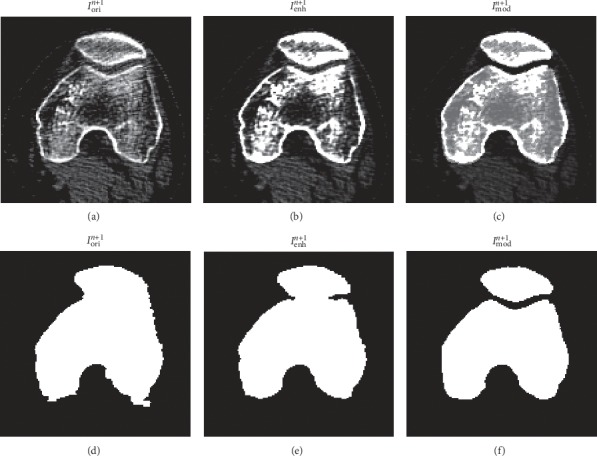
Effects of the contrast enhancement and the bone regions prediction on segmentation. An original CT image is shown in (a). It is modified by the contrast enhancement and further by the bone regions prediction, leading to the images in (b) and (c), respectively. Segmentation results on (a), (b), and (c) are shown in (d), (e), and (f), respectively, by the C-V models.

As an example, we show an original CT image in Figure 7(a), the image modified by the contrast enhancement in Figure 7(b), and the image modified further by the bone regions prediction in Figure 7(c). We observe in Figure 7(c) that the bone region prediction leads to improved intensity homogeneity of the bone regions, suppressed soft tissue intensities, and well cleared sutura between the bone regions. Applying C-V models on the three images, we obtain the corresponding segmentation results as shown in Figures 7(d), 7(e), and 7(f), respectively. Comparing these three figures, we observe that the proposed contrast enhancement and bone regions prediction techniques lead to significantly improved segmentation results.

## 4. Automatic Measurement

In a segmented CT image, we expect to have two major regions with right shapes, corresponding to the femur and the patella, respectively. In rare cases, it may happen that more or less than two regions are segmented on a CT image or/and the shapes of segmented bone regions change tremendously between CT images, mostly due to low CT image quality. These cases can be easily detected based on the number of and the geometric properties (*e.g.*, position and area) of the segmented regions. We simply discard these outlier cases and do not use them for measurement.

The CT images are acquired on parallel cross sections of the knee region, as shown in Figure 1. As such, we locate a few key points on each CT image on the boundaries of the femur region and the patella region, respectively, and then compute the central planes for the femur and the patella bones by optimally fitting those key points on the CT images.

### 4.1. Selection of Key Points

**Figure 8:**
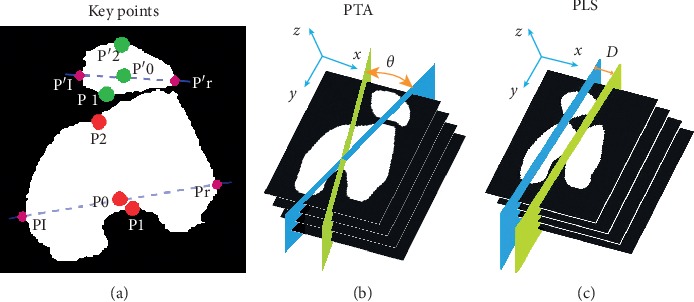
Key points, PTA, and PLS. As shown in (a), each bone has three key points selected: two central valley or peak points along the boundary and the middle of the leftmost and the rightmost boundary points (colored in purple). Key points of the two bone regions are colored in red and green, respectively. The definition of patella tilt angle (PTA) is illustrated in (b), which is the angle, *θ*, between the femur and the patella's central planes which are colored in yellow and blue, respectively. The definition of patella lateral shift (PLS) is illustrated in (c), which is the distance, *D*, between the parallel approximate central planes of the femur and the patella.

For the femur region in each CT image, we select three points as the key points: the two central valley points along the boundary and the middle of the leftmost and the rightmost points. For the patella region in each CT image, we select three points as the key points: the two central peak points along the boundary and the middle of the leftmost and the rightmost points. These key points can be easily identified through boundary tracking and inflection point detection. This key point selection scheme is illustrated in Figure 8(a).

### 4.2. Plane Fitting

The central plane of the femur (patella) bone is determined by optimally fitting a plane to the key points of the femur regions (patella regions) on the stack of CT images. In general, denoting the points as *p*_*i*_(*x*_*i*_, *y*_*i*_, *z*_*i*_) (*i*=1,2,…, *K*) and the plane equation as *z*=*ax*+*by*+*c*, the plane that optimally fits those points can be obtained by

(5) $$\begin{array}{c} {\text{argmin}}_{a,b,c}\overset{K}{\underset{i=1}{\sum }}{\left(z_{i}-ax_{i}-by_{i}-c\right)}^{2}, \end{array}$$

which can be solved with the least squares method.

### 4.3. PTA and PLS Measurement

We measure the patella tilt angle, *θ*, between the femur and the patella's central planes, as illustrated in Figure 8(b). It is measured by the angle between the normals of the two bone's central planes.

Further, we measure the patella lateral shift, *D*, between a pair of parallel approximate central planes of the femur and the patella, as illustrated in Figure 8(c). For this purpose, we fit a pair of parallel planes to the femur regions' and the patella regions' key points, respectively, and measure the distance, *D*, between the planes. Assuming that the equations of the two parallel planes are *z*=*ax*+*by*+*c*_1_ and *z*=*ax*+*by*+*c*_2_, given the femur regions' key points as *p*_*i*_(*x*_*i*_, *y*_*i*_, *z*_*i*_) (*i*=1,2,…, *K*) and the patella regions' key points as *p*_*i*_′(*x*_*i*_′, *y*_*i*_′, *z*_*i*_′) (*i*=1,2,…, *K*), the parallel plane fitting is done by

(6) $$\begin{array}{c} {\text{argmin}}_{a,b,c_{1},c_{2}}\overset{K}{\underset{i=1}{\sum }}{\left(z_{i}-ax_{i}-by_{i}-c_{1}\right)}^{2}+{\left(z_{i}^{\prime }-ax_{i}^{\prime }-by_{i}^{\prime }-c_{2}\right)}^{2}, \end{array}$$

using the least squares method.

## 5. Results and Discussion

In the experiments, we conduct automatic segmentation and measurement on our dataset of knee CT images using the proposed scheme and validate the results of both the segmentation and the measurement.

### 5.1. Dataset

Our dataset is composed of fifteen patients' knee CT images that were acquired using the Toshiba Aquilion ONE CT scanner in the affiliated hospital of Shandong University of TCM. Among the fifteen patients, ten are female and five are male. While being scanned, each patient was asked to move her/his legs freely from 0° to about 90°, and 22 CT image sequences were sampled at 22 time instances, one at each, during the scanning process. Each CT image sequence includes 320 images, 70 of which corresponding to the upper part of the leg (ref. Figure 1(a)) are used as the input to our system. The CT scanner is set up such that the thickness of each slice and the interval between two adjacent slices are both 0.5 mm, the default window width is 30 HU, the window level is 320 HU, and every CT image has a resolution of 512 × 512.

### 5.2. Validation of Bone Region Segmentation

In this section, we validate the bone region segmentation results both visually and quantitatively. In order to validate our choice of the C-V models [17], we compare with the following benchmark methods for image segmentation: the bias-corrected fuzzy c-means method (BCFCM) proposed by Mohamed et al. [35], the updated region-based active contour method using region-scalable fitting (RSF) energy function proposed by Li et al. [18], the level set method with bias field (LSEBFE) proposed by Li et al. [21], and the active contours driven by local image fitting energy (LIF) proposed by Zhang et al. [22]. For each image segmentation method, we run it both without and with our proposed framework, meaning that we run it both directly on the original CT images and on the CT images after modification with the approach proposed in Sections 3.1 and 3.2.

BCFCM modifies the objective function of the standard fuzzy c-means (FCM) algorithm to compensate for intensity inhomogeneities and allows the labeling of a pixel (voxel) to be influenced by the labels in its immediate neighborhood, which leads to better segmentation results than the standard FCM. RSF is a modified region-based active model using local intensity information at a controllable scale, which can preserve local details better and have higher robustness to intensity inhomogeneity. Note that BCFCM and RSF have been widely used in medical image segmentation. LSEBFE is a region-based level set method with bias field. It derives a local intensity clustering property of the image intensities and defines a local clustering criterion function, which are integrated with respect to the neighborhood center to give a global criterion of image segmentation. This criterion defines an energy in terms of the level set functions and a bias field that accounts for the intensity inhomogeneity of the image. It is more robust to initialization, faster, and more accurate than the well-known piecewise smooth model. LIF is a region-based active contour model that embeds the image local information. It uses Gaussian filtering for variational level set to regularize the level set function. It can not only ensure the smoothness of the level set function but also eliminate the requirement of reinitialization. Both LSEBFE and LIF are proposed to segment images with intensity inhomogeneities.

#### 5.2.1. Visual Validation

**Figure 9:**
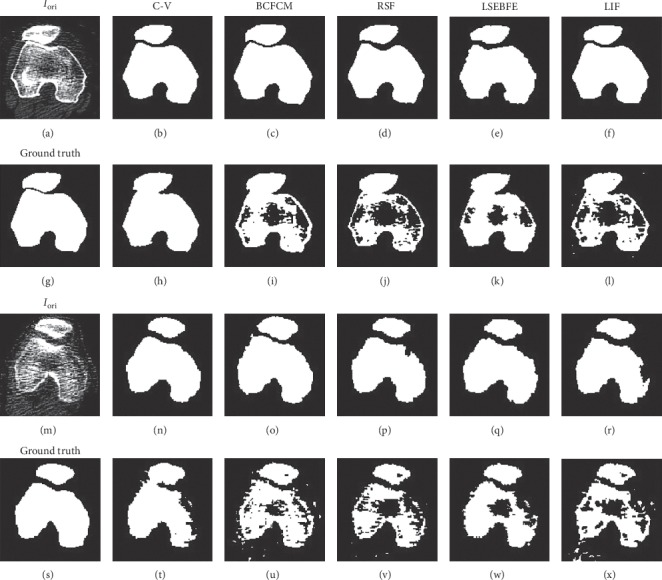
Segmentation results by different image segmentation methods on two representative challenging CT images. The first column shows the original CT images and the ground truth of their segmentations, and the following columns show the segmentation results by C-V, BCFCM, RSF, LSEBFE, and LIF, respectively. The segmentation results in the first and the third rows are obtained with our proposed framework while those in the second and the fourth rows are obtained without our framework.

In this section, we present the segmentation results of C-V, BCFCM, RSF, LSEBFE, and LIF on two representative challenging CT images, as shown in Figure 9. In the first image (in Figure 9(a)), the two bone regions are very close to each other while in the second image (in Figure 9(m)), there is more significant noise and weaker bone boundary response. Besides, both images have a high level of intensity inhomogeneity. In Figure 9, the first column shows the original CT images and the ground truth of their segmentations provided by experienced clinicians (*i.e.*, Jiushan Yang, Shaoshan Wang, and Ruiqi Zou), and the following columns show the segmentation results by the five image segmentation methods, respectively. Further, the segmentation results in the first and the third rows are obtained with our framework (*i.e.*, CT image modification followed by image segmentation) while those in the second and the fourth rows are obtained without our framework (*i.e.*, they are obtained by direct image segmentation).

Comparing the segmentation results with and without our framework in Figure 9, we observe that for any of the image segmentation methods, our proposed framework promotes the performance by a large margin, leading to more neatly and accurately segmented femur and patella regions. This also demonstrates the robustness of the proposed framework to soft tissues responses, intensity inhomogeneities, and noises in the CT images. Comparing the segmentation results of all the five image segmentation methods with our framework, we observe that the C-V method is the most advantageous in terms of accuracy and smoothness of segmented bone boundaries, confirming our choice of the C-V method in the proposed scheme.

#### 5.2.2. Quantitative Validation

For the quantitative validation, we randomly choose the automatic segmentation results on 30 CT images from each leg of each patient's dataset and also manually mark the bone regions segmentation on each chosen CT image which is used as the ground-truth reference. Similar to Yao et al. [36], we use three metrics, *i.e.*, overlap rate (OLR), false-positive rate (FPR), and Dice similarity coefficient (Dice), to quantitatively validate the segmentation accuracy. On a CT image, if we denote the automatic and the ground-truth segmentations of a bone region as *R*_*a*_ and *R*_*g*_, respectively, these metrics are defined as OLR=|*R*_*a*_∩*R*_*g*_|/|*R*_*g*_| × 100%, FPR=|*R*_*a*_ − *R*_*g*_|/|*R*_*a*_| × 100%, and Dice=2 × |*R*_*a*_∩*R*_*g*_|/|*R*_*a*_|+|*R*_*g*_|. In addition, we measure the separation rate, SR, of the patella and the femur regions in the segmentation result. If they are completely separated, we set SR=100%; otherwise, SR=0%.

In Table 1, we show the mean and standard deviation statistics of OLR, FPR, and Dice and the mean statistics of SR for all the five image segmentation methods (C-V, BCFCM, RSF, LSEBFE, and LIF) with and without our framework (*i.e.*, CT image modification followed by binary image segmentation) on all the 30 × 2 × 15 test CT images. Here, OLR, FPR, and Dice metrics are computed by treating the patella and the femur regions as one united bone region in each CT image.

From Table 1, we observe that (1) for any of the five methods, its mean OLR, mean Dice, and mean SR values are all increased and its mean FPR value is decreased when our framework is used, showing the effectiveness of our proposed CT image modification technique; (2) for any of the five methods, its mean SR value with our framework reaches 100%, showing the effectiveness of our proposed bone regions prediction technique in separating the two bone regions; and (3) when used with our framework, the C-V method yields the largest mean Dice, a SR value of 100%, the second largest mean OLR, and the third smallest mean FPR value and appears superior to the other methods considering all metrics overall.

In Table 2, we show the overlap rate and false-positive rate statistics for the femur and the patella regions on both legs of all the patients. For each bone region on each leg of each patient, we compute the two rates on all the 30 chosen CT images, average the rates over the 30 samples, and list the average in Table 2 where OLR_*F*_ (OLR_*P*_) and FPR_*F*_ (FPR_*P*_) mean the overlap rate and the false-positive rate of the femur (patella) region, respectively. Further, we compute the mean and the standard deviation on each of the OLR_*F*_, OLR_*P*_, FPR_*F*_, and FPR_*P*_ statistics of each leg and place them at the bottom two rows in Table 2.

From Table 2, we observe that (1) for either leg and either bone region, the mean overlap rate is close to 95% and the mean false positive rate is close to 2%, showing the high accuracy of our bone regions segmentation scheme; (2) the standard deviations of the various rate statistics are all below or slightly above 3%, showing the stability and robustness of our bone regions segmentation scheme; and (3) rates of the same type on both legs are quite comparable, again confirming the stability and robustness of our bone regions segmentation scheme.

We show the Dice statistics for the femur and the patella regions on both legs of all the patients in Table 3. For each bone region on each leg of each patient, we compute the Dice on all the 30 chosen CT images, average them over the 30 samples, and list the average Dice in Table 3.

From Table 3, we see that all the Dice statistics are above or slightly below 0.96 and the standard deviations of the Dice coefficient are close to 0.02, further confirming the high accuracy, stability, and robustness of our bone region segmentation scheme.

### 5.3. Validation of PTA and PLS Measurement

#### 5.3.1. Measured Angles and Distances

In this validation, we pick up the CT image sequences taken at four random time instances, *T*_1_, *T*_2_, *T*_3_, and *T*_4_, for four randomly picked patients' left or right legs. For the CT image sequence at each time instance, we use our system to automatically measure the angle (*i.e.*, the PTA), *θ*, and the distance (*i.e.*, the PLS), *D*, between the two bones' central planes. Note that when *θ* > 15°, we do not measure *D*. For the purpose of comparison, we ask several radiologists to measure the same parameters on the CT images manually. We use the unit of degree for angle measurement and the unit of millimeter for distance measurement. Note that for the automatic measurement, we have converted the unit of pixel to the unit of millimeter, knowing that one pixel corresponds to 0.95 millimeters in the photographing. The corresponding statistics is given in Table 4, from which we see that there is very little difference between the automatically and the manually measured angle numbers. Similarly, the automatically and the manually measured distances also closely match each other.

#### 5.3.2. Diagnosis by Measured Results

We further test the accuracy and reliability of using the automatically measured results for diagnosis. According to orthopedists, the angle, *θ*, between the femur and the patella bones' central planes provides the most important basis for patellar dislocation diagnosis. Thus, patellar dislocation may be straightforwardly diagnosed by comparing the measured angle against threshold values. Specifically, as an initial test, we set our system to automatically diagnose normal if *θ* ≤ 10°, patellar subluxation if 10° < *θ* < 30°, and patellar dislocation if *θ* ≥ 30°.

**Figure 10:**
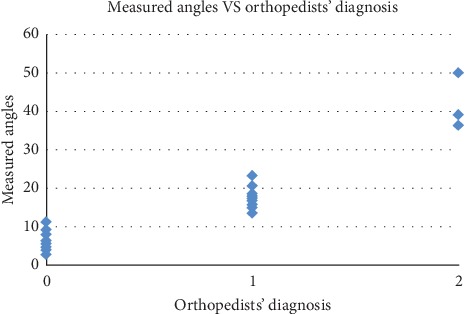
Distribution of automatically measured angles with respect to orthopedists' diagnosis: 0—normal, 1—patellar subluxation, and 2—patellar dislocation.

For this test, we have the dataset of 30 legs from 15 patients. For each leg, we use all the 22 CT image sequences and the average results of the 22 image sequences to make the diagnosis. Among the 30 samples, 11 samples (36.7%) are diagnosed as normal, 16 samples (53.3%) as patellar subluxation, and 3 samples (10%) as patellar dislocation, based on the automatically measured angles and the above-described diagnosis rule. On the same set of data, our orthopedists made diagnosis as well through manual measurement and clinic analysis and diagnosed 10 samples (33.3%) as normal, 17 samples (56.7%) as patellar subluxation, and 3 samples (10%) as patellar dislocation. Comparing the automatic and the manual diagnosis results, we find that the error rates of the automatic diagnosis on normal, patellar subluxation, and patellar dislocation are 9.1%, 7.1%, and 0%, respectively. Further, we visualize the distribution of the automatically measured angles with respect to the orthopedists' manual diagnosis results in Figure 10. We see from Figure 10 that all the cases with *θ* ≥ 30° are diagnosed by the orthopedists as patellar dislocation, and the majority of the cases with *θ* ≤ 10° and 10° < *θ* < 30° are diagnosed by the orthopedists as normal and patellar subluxation, respectively. There is fuzziness only for a small portion of cases with *θ* closely around 10°.

**Figure 11:**
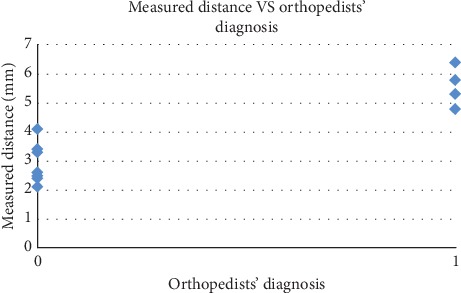
Distribution of automatically measured distances with respect to orthopedists' diagnosis: 0–normal and 1–patellar subluxation. The distance corresponds to the cases with measured angles between 5° and 15°.

As a refined test, we further investigate the effectiveness of using distance as an auxiliary means for the patellar dislocation diagnosis. We only focus on the samples with 5° < *θ* < 15°, as there is fuzziness for samples with *θ* around 10° in our initial test. For these samples, the distances between the two bones' central planes are automatically measured and their distribution with respect to the orthopedists' diagnosis results are plotted in Figure 11. From Figure 11, we see that a distance threshold of 4.5 mm will accurately separate the cases of normal and patellar subluxation, thus eliminating the errors of diagnosis in our initial test where only angles are used.

## 6. Conclusions

In this work, we have developed a system for automatic segmentation and measurement on knee CT images. Firstly, on each CT image in an input sequence, we segment the femur and the bone regions; thereafter, we identify key points on the bone regions' boundaries and conduct optimal fitting to obtain the central planes of the two bones; finally, angles and distances between the central planes are measured which can be used to assist doctors in patellar dislocation diagnosis.

Of the whole process, the biggest challenges exist with the bone region segmentation, due to the confusion from soft tissue responses and noises, inhomogeneity of bone region intensities, and close or even fused bone regions in the sutura area. To overcome these challenges, novel and effective methods are proposed to improve the quality of input CT images by enhancing the contrast of each CT image and predicting the bone regions in a CT image utilizing the coherence between adjacent CT images. The improved CT images are finally segmented using a region-based active contour method. The accuracy and robustness of the automatic segmentation and measurement results are validated in our experiments.

In the future, we will extend our system to measure more parameters as needed for the diagnosis. Furthermore, we will investigate reconstructing a 3D volume of the bones from the CT images and conduct measurements on this 3D volume with increased capability and flexibility.

## Figures and Tables

**Table 1:** Mean (MEAN) and standard deviation (SDEV) statistics of overlap rate (OLR), false-positive rate (FPR), and Dice similarity coefficient (Dice) and mean statistics of separation rate (SR) for different image segmentation methods (C-V, BCFCM, RSF, LSEBFE, and LIF) with and without our framework on all the 30 × 2 × 15 test CT images.

With our framework?	Methods	Performance metric			
		OLR	FPR	Dice	SR
Yes	C-V	95.09 ± 3.24	2.04 ± 2.70	0.965 ± 0.022	100.00
	BCFCM	99.12 ± 2.48	6.32 ± 5.63	0.962 ± 0.035	100.00
	RSF	93.69 ± 4.34	1.79 ± 2.33	0.958 ± 0.025	100.00
	LSEBFE	94.60 ± 3.96	1.85 ± 2.09	0.963 ± 0.023	100.00
	LIF	94.12 ± 3.83	9.43 ± 10.74	0.919 ± 0.063	100.00

No	C-V	95.04 ± 3.90	3.24 ± 3.43	0.958 ± 0.026	25.33
	BCFCM	81.88 ± 10.01	6.47 ± 12.03	0.862 ± 0.081	54.67
	RSF	69.66 ± 9.43	3.05 ± 3.81	0.807 ± 0.069	68.67
	LSEBFE	82.64 ± 7.55	2.76 ± 4.19	0.892 ± 0.055	55.33
	LIF	73.79 ± 7.13	11.18 ± 9.53	0.802 ± 0.062	68.67

Numeric entries for OLR, FPR, and Dice in the table are in the form of MEAN ± SDEV.

**Table 2:** Statistics of overlap rate (%) and false-positive rates (%) for the femur and the patella regions on both legs of the 15 patients.

Patient number	Left leg				Right leg			
	OLR_*F*_	FPR_*F*_	OLR_*P*_	FPR_*P*_	OLR_*F*_	FPR_*F*_	OLR_*P*_	FPR_*P*_
1	96.68	2.33	97.61	1.85	98.02	4.14	98.55	6.00
2	94.22	0.23	95.38	2.92	88.89	3.83	92.24	13.31
3	96.13	0.08	95.91	2.61	97.79	1.13	95.67	1.43
4	97.40	1.51	88.14	0.37	97.31	0.63	92.92	0.09
5	95.83	0.65	95.74	5.10	94.18	0.56	96.40	11.25
6	98.72	0.96	93.17	0.57	98.39	0.96	94.12	0.24
7	96.60	8.68	90.49	1.47	96.71	6.45	90.19	1.11
8	86.15	0.99	97.88	4.55	96.94	0.63	96.32	3.77
9	95.06	0.16	91.62	1.29	96.35	0.99	95.69	2.21
10	95.45	1.79	92.47	2.82	81.84	3.85	93.39	1.36
11	96.73	2.72	94.61	3.51	91.15	2.38	91.58	8.15
12	95.92	0.76	92.70	0.16	94.53	0.76	93.32	2.86
13	97.16	1.39	95.09	0.66	94.28	3.11	96.24	4.10
14	95.63	0.32	94.51	1.35	96.36	1.03	95.98	1.40
15	97.19	1.03	90.62	0.12	97.57	2.62	88.10	0.00
MEAN	95.66	1.57	93.73	1.96	94.69	2.21	94.05	3.82
SDEV	2.91	3.15	2.66	2.09	4.53	2.89	2.67	5.18

OLR = overlap rate, FPR = false-positive rate, *F* = femur, *P* = patella, and SDEV = standard deviation.

**Table 3:** Statistics of Dice similarity coefficient for the femur and the patella regions on both legs of the 15 patients.

Patient number	Left leg		Right leg	
	Dice_*F*_	Dice_*P*_	Dice_*F*_	Dice_*P*_
1	0.9716	0.9788	0.9692	0.9614
2	0.9691	0.9616	0.9235	0.8857
3	0.9798	0.9660	0.9833	0.9708
4	0.9794	0.9349	0.9833	0.9628
5	0.9756	0.9531	0.9674	0.9220
6	0.9888	0.9619	0.9871	0.9686
7	0.9358	0.9432	0.9490	0.9422
8	0.9211	0.9662	0.9813	0.9628
9	0.9739	0.9493	0.9766	0.9672
10	0.9680	0.9477	0.8839	0.9594
11	0.9699	0.9552	0.9425	0.9161
12	0.9754	0.9613	0.9682	0.9519
13	0.9787	0.9717	0.9550	0.9601
14	0.9761	0.9652	0.9764	0.9727
15	0.9807	0.9501	0.9742	0.9362
MEAN	0.9696	0.9577	0.9614	0.9493
SDEV	0.0204	0.0166	0.0288	0.0309

Dice = Dice similarity coefficient, *F* = femur, *P* = patella, and SDEV = standard deviation.

**Table 4:** Geometric measurement results on four patients' left or right legs at four time instances (*T*_1_, *T*_2_, *T*_3_, and *T*_4_).

Patient number	Type	Method	*T*_1_	*T*_2_	*T*_3_	*T*_4_
2_*l*_	Angle	Automatic	2.1	4.4	1.6	2.0
		Manual	2.0	4.5	1.5	2.0
	Distance	Automatic	0.6	0.7	0.7	0.3
		Manual	0.5	0.7	0.6	0.3

5_*r*_	Angle	Automatic	6.9	4.5	8.1	8.8
		Manual	6.5	4.3	7.8	8.9
	Distance	Automatic	2.3	2.4	3.3	3.7
		Manual	2.4	2.2	3.2	3.5

10_*l*_	Angle	Automatic	18.2	17.3	19.5	18.2
		Manual	18.4	17.5	19.2	18.2
	Distance	Automatic	7.9	8.3	7.5	8.0
		Manual	7.7	8.0	7.7	8.1

14_*r*_	Angle	Automatic	38.0	39.8	39.4	38.5
		Manual	38.5	40.3	39.2	38.9
	Distance	Automatic	—	—	—	—
		Manual	—	—	—	—

The units of degree and millimeter are used for angle and distance, respectively.

## Data Availability

The data used to support the findings of this study are available from the corresponding author upon request.

## Abbreviations

CT: Computerized tomography
CAD: Computer-aided diagnosis
PTA: Patellar tilt angle
PLS: Patellar lateral shift
C-V: Chan–Vese
BCFCM: Bias-corrected fuzzy c-means method
RSF: Region-scalable fitting
FCM: Fuzzy c-means
LSEBFE: Level set method with bias field
LIF: Local image fitting
OLP: Overlap rate
FPR: False-positive rate
Dice: Dice similarity coefficient
SR: Separation rate.
